# A Rare *De novo* Complex Chromosomal Rearrangement (CCR) Involving Four Chromosomes in An Oligo-asthenosperm Infertile Man

**Published:** 2014-10-04

**Authors:** Saba Asia, Hamed Vaziri Nasab, Marjan Sabbaghian, Hamid Kalantari, Shabnam Zari Moradi, Hamid Gourabi, Anahita Mohseni Meybodi

**Affiliations:** 1 Department of Genetics at Reproductive Biomedicine Research Center, Royan Institute for Reproductive Biomedicine, ACECR, Tehran, Iran; 2 Department of Andrology at Reproductive Biomedicine Research Center, Royan Institute for Reproductive Biomedicine, ACECR, Tehran, Iran

**Keywords:** Complex Chromosomal Rearrangement (CCR), Infertility, Karyotype, FISH

## Abstract

Complex chromosomal rearrangements (CCRs) are rare events involving more than two chromosomes and over two breakpoints. They are usually associated with infertility or sub fertility in male carriers. Here we report a novel case of a CCR in a 30-year-old oligoasthenosperm man with a history of varicocelectomy, normal testes size and normal endocrinology profile referred for chromosome analysis to the Genetics unit of Royan Reproductive Biomedicine Research Center. Chromosomal analysis was performed using peripheral blood lymphocyte cultures and analyzed by GTG banding. Additional tests such as C-banding and multicolor fluorescence in situ hybridization (FISH) procedure for each of the involved chromosomes were performed to determine the patterns of the segregations. Y chromosome microdeletions in the azoospermia factor (AZF) region were analyzed with multiplex polymerase chain reaction. To identify the history and origin of this CCR, all the family members were analyzed. No micro deletion in Y chromosome was detected. The same *de novo* reciprocal exchange was also found in his monozygous twin brother. The other siblings and parents were normal. CCRs are associated with male infertility as a result of spermatogenic disruption due to complex meiotic configurations and the production of chromosomally abnormal sperms. These chromosomal rearrangements might have an influence on decreasing the number of sperms.

## Introduction

Complex chromosomal rearrangements (CCRs) are balanced or unbalanced structural aberrations that are characterized by three or more breakpoints, located on more than two chromosomes (1). CCRs are very rare events in the human population (2). Only about 250 patients with CCRs have been reported in the literature, however, this number will likely increase owing to the application of molecular cytogenetic techniques (3). The balanced CCRs range from simple three-way exchanges between three chromosomes to highly complex translocations involving four or ﬁve chromosomes with multiple breaks, inversions, and insertions. Up till now, CCRs have been classiﬁed according to whether they are inherited or *de novo*, and according to the number of chromosomes or the number of breaks involved (4). In almost 70% of the cases (2), especially in most of the familial cases, the phenotype is normal in the apparently balanced carriers but they may have a signiﬁcant risk of reproductive failure (2, 5). Most *de novo* CCRs originate from spermatogenesis and cause mental retardation in high incidence, whereas most familial CCRs are of maternal origin and usually have three to four breakpoints (6-9). Most of the female patients with CCRs have been identiﬁed because of giving birth to malformed children or having repeated spontaneous abortions (9), while vast majority of the males with CCRs have been found in men showing infertility problems (10). According to the literature, the complexity of meiotic conﬁgurations may cause hypospermatogenesis or spermatogenic arrest in CCR-carrying patients (2). A review of published reports revealed that 13.7% of azoospermic men and 4.6% of oligozoospermic men have an abnormal karyotype. In the azoospermic group, sex chromosome abnormalities predominate, mainly 47, XXY. In the oligozoospermic group, autosome anomalies, such as Robertsonian and reciprocal translocations, are the most frequent karyotypic abnormalities (11).

The interpretation of CCRs by conventional banding techniques alone may be impossible, particularly when deletions, insertions or inversions as well as reciprocal translocations occur simultaneously. Fluorescent in-situ hybridization (FISH) with chromosome-speciﬁc DNA probes allows exploring chromosome rearrangements in greater detail and is a useful tool for an accurate diagnosis (12).

The present case represents a new case of CCR in a man with spermatogenic defect. The CCR of this oligozoospermic male involves four chromosomes (13, 14, 16 and 18) and five breakpoints.

## Case report

A 30-year-old man suffering from infertility for three years underwent cytogenetic examination. For this research, a written informed consent for use of the results of examinations was obtained from the patient. There were no mental retardation, no malformation, no gynecomastia, no erectile dysfunction, no thromboembolic disease and no reduced muscle strength. Testes volumes were normal. His parents were not related. Although, two of his brothers had children, his monozygotic twin brother was also infertile. Serologic analysis revealed serum levels of FSH, LH, prolactin, and testosterone were in normal ranges. Clinical assessment verified the presence of varicocele grade-I on the left side. The semen analysis indicated total volume (3.7 ml), normal *pH* (7.8), low concentration (0.6 millions/ml) and normal color (whitegray). It also showed reduced sperm motility; sperm total motility (5%) together with a low yield of progressively motile sperm and teratozoospermia (98% of spermatozoa showed abnormal morphology) and low viability (44%). Histological assessment of testis biopsy specimen showed incomplete spermatogenic arrest with signs of sloughing. Few spermatozoa were seen. For more investigation, cytogenetic tests were proposed (13). Cytogenetic analysis was performed according to standard methods on phytohemagglutinin (PHA)-stimulated peripheral lymphocyte cultured cells from the patient, his brother, his sister and his parents. Briefly, cells were cultured in complete RPMI 1640 (GIBCO) for 72 hours. The Colcemid arrested cells were spun and the pellet was resuspended in 5-10 ml hypotonic solution for about 20 minutes at 37˚C. After centrifugation the cells were fixed with Carnoy’s fixative. Fixed cells were used for slide preparation. The procedure was followed by FISH, GTG as well as C-banding techniques.

At least 20 GTG banded metaphases from the cases were analyzed at a resolution of 550 bands. The latest ISCN guidelines for chromosome nomenclature were followed (14).

**Fig 1:**
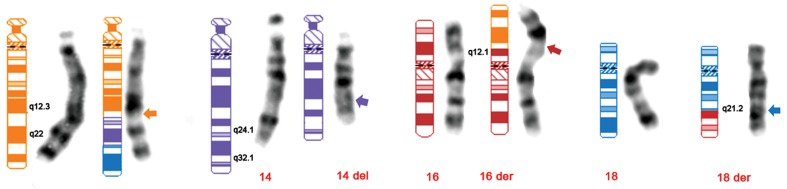
Idiogram and GTG banded of involved chromosomes in this CCR. Arrows indicate the breakpoints.

Karyotype of peripheral blood revealed a *de novo* complex chromosomal rearrangement; 46, XY, der(13) t(13; 18) (q22; q21.2) ins(13;14) (q22; q24q32.1), del(14) (q24q32.1), der(16) t(16; 13) (p12.3; q22), der(16; 18) (p12.3; q21.2) (Fig 1).

**Fig 2:**
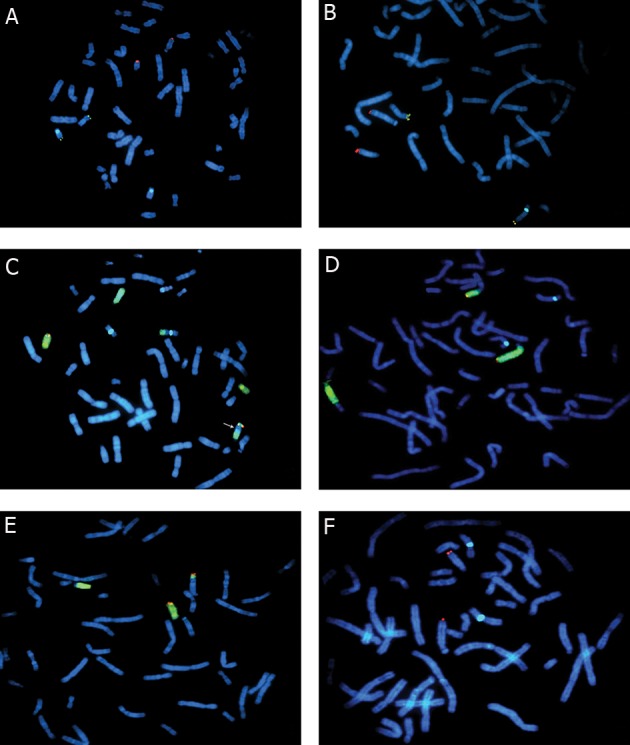
FISH of mitotic lymphocytes with whole chromosome or segmental paints: (A and B) Chr 18 Centromere probe (Aqua), Chr 14q Telomere probe (Orange), 16p Telomere probe (Green), C. 16 Centromere probe (Aqua), 18q Telomere probe (Orange), chromosome 13 and 18 paint (Green). The arrow shows the inserted segment from 14q to 13q, D. Chr 13 paint (Green), 13q Telomere probe (Orange), 18 Centromere probe (Aqua), E. Chr 18 paint (Green), 18q Telomere probe (Orange), F. 16 Centromere probe (Aqua), 14q Telomere probe (Orange).

According to the karyotype and judging from figure 2, the following is supposed to have happened: a segment of 14q (bands between q24 and q32.1) has inserted to the long arm of chromosome 13 at band q22. A segment from the same chromosome 13 from q22 has moved to the short arm of 16(p12.3), the segment from 16p has moved to the long arm of 18 at band q21.2 and the segment from 18 has moved to the long arm of 13. This would be a CCR involving four chromosomes with five breakpoints.

Additional multicolor fluorescence in situ hybridization (FISH) procedure on metaphase chromosomes was performed on prepared slides to determine the exact patterns of this CCR according to the standard cytogenetic protocols (15, 16). Appropriate DNA probes (Vysis, Abbott Molecular, USA) for involved chromosomes were applied. For this purpose, a welltuned fluorescent microscope (Olympus BX51, Japan) equipped with necessary and optimum filter sets (Spectrum Orange/Spectrum Green/ DAPI single band pass filter sets, Abbott Molecular, USA) and an image acquisition and processing software (Cytovision V4.0, Applied Imaging, Genetix, UK) were used (Fig 2).

His monozygotic twin brother had the same karyotype. De novo translocation was confirmed by the normal karyotype of the parents. His sister also had a normal karyotype.

The patient was then screened for Yq microdeletions. Genomic DNA was extracted from peripheral blood samples using the Genomic DNA Extraction Kit (Bioneer, Korea). Detection of microdeletions on the Y-chromosome was based on three multiplex PCRs (17). Molecular analysis showed no microdeletions in the Y chromosome.

## Discussion

Male infertility may be attributed to chromosomal alterations that usually involve the sex and autosomal chromosomes (18, 19). It had been reported that substantial cases of infertile men have constitutional chromosomal abnormalities, including 47, XXY, Robertsonian and reciprocal translocation of autosomes (11). Infertile men with spermatogenesis impairment are 10 times more likely to have structural chromosome abnormalities than the normal population (5.1% compared with 0.5% respectively) (20). As the role of a simple reciprocal translocation for male fertility has been well determined, CCRs can lead to more severe reproductive impairment (21). Infertility in such situations is usually related either to disturbance in meiosis or to the generation of unbalanced gametes through chromosome missegregation (20).

**Fig 3:**
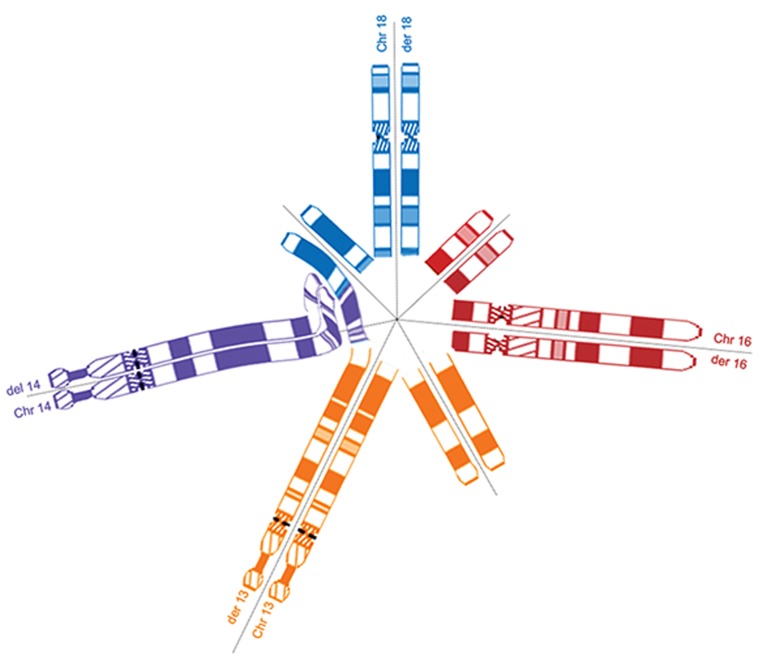
This schematic figure shows the heptavalent pachytene configuration adopted at meiosis-I by CCR. In this example, the configuration allows the efficient synapsis of the eight chromosomes involved in this complex.

The CCR presented here theoretically represents a heptavalent structure at meiosis I (Fig 3). It has been assumed that spermatogenic arrest occurs as a consequence of the complex meiotic configurations during meiosis (7). As few sperms were found in seminal analysis in this patient, it can be concluded that a deep impairment of spermatogenesis occurred at the late pachytene stage which resulted in cell death of most of the spermatocytes and eventually perturbation of spermiogenesis. Only few spermatocytes could escape pachytene apoptosis and were able to deal with a heptavalent in the metaphase I spindle and pass to anaphase I.

The origin of pachytene apoptosis has been attributed to a 'pachytene checkpoint' (22) which detects failures in chromosome synapsis and recombination. In the present case, the long stretches of asynaptic regions in the heptavalent at pachytene could initiate the apoptotic process. Such an explanation may justify the greatly reduced sperm parameters and severe oligozoospermia observed in this carrier. Interestingly, a lower frequency of unbalanced sperms than expected was found by Kirkpatrick and Ma in a carrier of a rare CCR. They hypothesized that a great amount of unbalanced chromosome complements are in fact produced during segregation but selection during spermatogenesis prefers spermatogonia which contains balanced/ normal chromosome complements. (7).

Cytogenetic studies (different banding techniques) and FISH were performed on this patient because he was supposed to undergo intracytoplasmic sperm injection (ICSI) procedure. As the risk of chromosome aberration transmission always exists in oligo- or azoospermic males, cytogenetic study prior to ICSI is of great importance (2). In the present case, the use of FISH technique was necessary for the correct diagnosis of this CCR. The availability of speci.c DNA probes and chromosomal libraries have made FISH clinically applicable. This has shown that CCRs may be more common than initially considered. Although it is suggested that the more complex a CCR is, the more severe the spermatogenic impairment will be, according to the literature, neither the origin and the complexity nor the number of breaks can be used to predict infertility (4, 23). Surprisingly, the normal phenotype of this patient suggests that the breakpoints in involved chromosomes do not inactivate functional genes or regions with regulatory functions, whose disruption could produce phenotypic alterations. Nevertheless, other disruptions at the molecular level cannot be disregarded. Spermatogenesis dysfunction in translocation carriers can now be bypassed by ICSI. However, ICSI is not considered a solution for infertility in male carriers of CCRs because of the low percentage of balanced sperm availability (3, 4, 24). Imbalanced sperms can lead to reproductive impairments such as fetal abnormalities and repeated spontaneous miscarriages. The incidence of having normal healthy babies in CCR carriers is thus very low. Nonetheless, CCR carriers still have a limited chance of having a healthy child (1).

Preimplantation genetic diagnosis (PGD) with FISH has been applied successfully to detect chromosomal imbalances in preimplantation embryos before being transferred into the mother’s uterus. Although PGD-FISH, due to the limited availability of FISH probes, is not efficient in diagnosing CCR-carrying embryos, it may be useful in selecting balanced embryos in such patients (1, 25).

This is another study that emphasizes the importance of probing techniques (e.g. FISH) as an ideal confirmatory method in cytogenetic studies. Moreover, this study also focuses on this fact that, although a vast majority of people with CCR, despite having a high extent of genetic alterations, may apparently seem normal, they are vulnerable to gametogenesis defect.
